# Comparative Analysis of Laboratory-Based and Spectroscopic Methods Used to Estimate the Algal Density of *Chlorella vulgaris*

**DOI:** 10.3390/microorganisms12061050

**Published:** 2024-05-23

**Authors:** György Fekete, András Sebők, Szandra Klátyik, Zsolt István Varga, János Grósz, Imre Czinkota, András Székács, László Aleksza

**Affiliations:** 1 Institute of Environmental Sciences, Hungarian University of Agriculture and Life Sciences, Páter Károly u. 1, H-2100 Gödöllő, Hungary; fekete.gyorgy@uni-mate.hu (G.F.); sebok.andras@uni-mate.hu (A.S.); klatyik.szandra@uni-mate.hu (S.K.); varga.zsolt.istvan@uni-mate.hu (Z.I.V.); grosz.janos@uni-mate.hu (J.G.); czinkota.imre@uni-mate.hu (I.C.); aleksza.laszlo@uni-mate.hu (L.A.); 2 Profikomp Environmental Technologies Inc., Kühne Ede u. 7, H-2100 Gödöllő, Hungary

**Keywords:** algal biomass, microalgae, magnesium content, chlorophyll a, UV–Vis spectrophotometry, optical density, fluorescence detection, cell count, cell weight, correlation scanning

## Abstract

*Chlorella vulgaris* is of great importance in numerous exploratory or industrial applications (e.g., medicals, food, and feed additives). Rapid quantification of algal biomass is crucial in photobioreactors for the optimization of nutrient management and the estimation of production. The main goal of this study is to provide a simple, rapid, and not-resource-intensive estimation method for determining the algal density of *C. vulgaris* according to the measured parameters using UV–Vis spectrophotometry. Comparative assessment measurements were conducted with seven different methods (e.g., filtration, evaporation, chlorophyll a extraction, and detection of optical density and fluorescence) to determine algal biomass. By analyzing the entire spectra of diluted algae samples, optimal wavelengths were determined through a stepwise series of linear regression analyses by a novel correlation scanning method, facilitating accurate parameter estimation. Nonlinear formulas for spectrometry-based estimation processes were derived for each parameter. As a result, a general formula for biomass concentration estimation was developed, with recommendations for suitable measuring devices based on algae concentration levels. New values for magnesium content and the average single-cell weight of *C. vulgaris* were established, in addition to the development of a rapid, semiautomated cell counting method, improving efficiency and accuracy in algae quantification for cultivation and biotechnology applications.

## 1. Introduction

Microalgae, the minuscule powerhouses of the aquatic and terrestrial environment, are revolutionizing various scientific disciplines with their diverse applications [1,2,3,4]. Despite their microscopic size, they play a critical role in Earth’s ecosystem and offer opportunities to solve global challenges [5,6,7,8]. Nearly 50% of the atmospheric oxygen originates from marine photosynthesis, predominantly driven by microalgae and cyanobacteria [9,10]. Various algae communities in aquatic ecosystems play an essential role in food webs and different nutrient transport processes [5,11,12,13]. Thus, algal density is a key parameter in water quality assessment as a descriptor for the rate of eutrophication [14,15], as well as in algal technologies as a parameter for successful cultivation rates.

Autotrophic algae communities significantly contribute to the primary production of biomass and oxygen [16,17,18]. In addition, they can bind and utilize a significant amount of CO_2_ and mitigate the adverse effects of climate change by reducing the level of greenhouse gases [19,20]. Different algae species are distributed all over the world, and they can adapt rapidly and grow exponentially in complex natural habitats and even in extreme conditions due to their morphological, physiological, and structural diversity [21,22,23,24,25]. However, several species are sensitive to toxicants; thus, various algae species are widely recommended for ecotoxicological assays to evaluate the toxicity of water contaminants or as bioindicators for water pollution [26,27,28,29,30]. Microalgae can be an effective tool during the removal of nutrients, heavy metals, and other pollutants from natural waterbodies and wastewaters [31,32,33,34,35]. Moreover, microalgae and cyanobacteria such as *Chlorella vulgaris* Beijerinck [36] and *Arthorspira platensis* Gomont (commercial name: *Spirulina platensis*) [37] are consumed as food supplements as well [38,39,40], and they are used in various industrial activities, such as in aquaculture and during the production of pharmaceuticals, animal feed, and cosmetics [41,42,43,44,45]. Furthermore, recent studies have shown that certain species of microalgae contain high contents of lipids, making them suitable for biofuel production [41,42,46]. However, differences can be observed in the biomolecular composition of various microalgae species, particularly concerning proteins, carbohydrates, and lipids. Their carbohydrate content, including starch, sugars, glucose, and other polysaccharides, can reach up to 64% of their biomass. Similarly, the proteins can reach up to 71%, while the lipids can comprise up to 22% of their biomass [47,48,49,50].

The unicellular green eukaryotic Chlorophyta microalga *C. vulgaris* has attracted great scientific and commercial interest for decades. It is one of the most frequently used algal test organisms in various environmental monitoring surveys and ecotoxicological studies, due to its easy maintenance under laboratory conditions and its ability to grow under a wide variety of climatic conditions [51]. *C. vulgaris* has a high content of proteins (51–58%, calculated as % of dry matter), carbohydrates (12–17%), and lipids (14–22%) [52,53]; thus, it has received broad attention due to its potential benefits in many industrial, (bio)technological, and food supplement applications [52,54]. Moreover, *Chlorella* also contains different vitamins, minerals, amino acids, and trace elements [55,56]. Regarding pigments, it contains both chlorophyll a (Chl a) and chlorophyll b, in addition to other carotenoids (e.g., β-carotene, antheraxanthin, and lutein) [57]. *C. vulgaris* can be easily cultivated in open ponds or photobioreactors on a large scale for several industrial uses. The main producers and consumer countries of *Chlorella* are Japan and Taiwan, with an annual production of nearly 3500 tons [58,59]. However, in 2021, the global market of *Chlorella* was dominated by Europe [60,61,62]. Since *C. vulgaris* uses CO_2_ for photosynthesis, its cultures are supplied with enriched air containing at 1–5% CO_2_ for rapid growth. However, excessively high levels of CO_2_ can inhibit growth or damage cells due to pH changes [19,63,64]. The growth rate and biomass production of algae communities, as well as the chemical composition of algae cells (cellular content), significantly depend on various environmental factors (e.g., illumination, temperature, and nutrients) [52,65,66,67,68]. Therefore, successful and effective algal biotechnology highly depends on the algal species selection and the specific culture conditions [69,70,71,72].

The discharge of untreated wastewater into aquatic environments contributes to severe water pollution, endangering both human health and aquatic ecosystems [73,74]. An economical and eco-friendly alternative to conventional wastewater treatment (CWWT) systems is the use of microalgae-based wastewater treatment [75,76]. In contrast to CWWT, the microalgae-based method is highly effective in the removal of nutrients (such as nitrogen and phosphorus compounds), heavy metals, organic micropollutants, and pharmaceuticals from wastewater, concurrently yielding economically viable biomass for bioenergy production [77,78,79,80]. Due to its special characteristics such as high nutrient uptake capacity, rapid growth rate, and adaptability to various wastewater conditions, *C. vulgaris* can be used for wastewater treatment (WWT) [81]. Through efficient bioaccumulation and biosorption, *C. vulgaris* can contribute to the filtration of wastewater in addition to the elimination of heavy metals (e.g., Cd, Cu, and Zn) and residues of pharmaceuticals [82,83,84,85,86,87]. Furthermore, *C. vulgaris* can be used as the raw material of biofuel, animal feed, cosmetics, and fertilizer production [43,52,88,89,90,91,92,93]. *C. vulgaris* is consumed as food supplements and used for medical treatment due to its health benefits (e.g., assumed anticarcinogenic and immunomodulatory effects) [58,94,95].

To optimize nutrient management, estimate the potential production of valuable components, and evaluate the growth and productivity of *C. vulgaris*, the determination of algal biomass is essential [96]. The accurate assessment of microalgal biomass is also crucial for the characterization of the efficacy and sustainability of *C. vulgaris* during the management of WWT and water pollution. In addition to the determination of alga biomass, the measurement of Chl a content is also useful during the evaluation of the effectiveness and productivity of *C. vulgaris* in WWT systems [97]. The algal biomass and Chl a concentration can be measured using various techniques, such as turbidimetry [98,99,100], spectrophotometry [101,102,103], fluorescence-based methods [104,105], and laser-based analytical methods [106]. The qualitative and quantitative determination of phytoplankton biomass is generally performed by time-consuming methods, like direct cell counts under a microscope or the measurements of cellular mass or volume. Spectrometric and imaging methods provide accurate and time-saving methods for the determination of algal biomass. The previously recommended spectroscopic methods with linear mathematic models for the monitoring of the algal biomass of *C. vulgaris* are summarized in Table 1.

According to the research of Griffiths et al., there is a lack of agreement in the literature regarding the optimal wavelength for cultivating microalgae [101]. Pigments absorb light more intensely in specific parts of the spectrum. Selecting a wavelength within the peak absorbance range of the pigment can provide the strongest signal but may also lead to the largest error if the pigment content in the cells varies. Despite this, using wavelengths within the maximal absorbance range of Chl a (400–460 nm and 650–680 nm) is commonly reported. An additional study highlights that the optimal wavelength is highly affected by the cell size and chlorophyll content, which are also dependent on the age of the cell and the age of the culture [114].

Nonetheless, monitoring of microalgae growth is difficult in bioreactors. The vertical and horizontal distributions of phytoplankton is highly affected by several factors including water temperature, underwater light conditions, UV radiation, and the availability of nutrients [115,116,117,118]. Therefore, several factors, such as controlled conditions (e.g., temperature, pH, and nutrient levels), adequate light wavelengths at different depths, and minimal algae overlap, play a crucial role in optimal microalgae cultivation and biomass production in bioreactor systems [96,119,120,121,122]. To avoid competition for light, nutrients, and space, which may limit the growth of the preferred microalgae species, minimizing algae overlap is essential for the maintenance of the optimal bioreactor conditions (e.g., temperature, pH, and nutrient levels) to maximize microalgae growth and biomass production for various industrial applications. The analysis of microalgal growth presents several challenges during the cell counting and drying steps of analytical procedures due to the high laboratory requirements, time-consuming procedures, and the potential for sample destruction [123]. Biological models indicate the complex nonlinear dynamic behavior of algal growth [124]. Due to the development of the initial biological models, the robustness and the sensitivity of the models against parameter uncertainties improved, during the comparisons of experimental data with the model dynamics of microalgal growth [124]. However, modeling the dynamics of microalgal growth is not easy due to the persistent nonstationary behavior and a complex feedback system that occurs from the population level to the cell level through light attenuation [125].

The primary goal of this study was to provide a simple, fast, and not-resource-intensive estimation method for the determination of the selected measurement parameters (e.g., magnesium (Mg) content, Chl a content, cell count) characterizing the algal biomass of *C. vulgaris* through UV–Vis spectrophotometry. To allow comparative assessment of the UV–Vis spectrophotometric method, algal biomass was determined by seven different methods, such as filtration, evaporation, Chl a extraction, Mg concentration, cell count, optical density, and fluorescence detection on a dilution series prepared from a concentrated algae culture of *C. vulgaris*. Based on the detected entire spectra of each diluted algae sample, linear regression was used for the determination of the optimal wavelengths ensuring the strongest correlation for the given parameters. Thus, these determinations are capable of proposing, for each measured parameter, where it should be measured in the UV–Vis wavelength range and what relationship (mathematical formula) should be used for the estimation. A secondary objective was to develop a universal formula for estimating biomass concentration and ensure guidance on selecting appropriate measuring devices based on the different levels of algae concentrations. An additional objective of our study is to provide updated information on the physical characteristics of *C. vulgaris*. Therefore, the average single-cell weight and the Mg content of this algae strain were also established.

## 2. Materials and Methods

### 2.1. Microalgae and Strain Culture Conditions

During the measurements, a dilution series was prepared from a stock culture of *C. vulgaris.* The applied unicellular, green algae strain (*C. vulgaris* Beijerinck—CCAP 211/11b) was obtained from the Culture Collection of Algae of Charles University in Prague (Prague, Czech Republic) and cultured in Scharlau Algae Broth (Art. No. 02-007) nutrient salt (Scharlab group, Barcelona, Spain). The selected algae species has very little sensitivity to environmental changes, tolerates the elevated carbon dioxide concentrations during the breeding process well, and is easy to handle. A very extensive amount of literature is available connected to *C. vulgaris*, and there is a huge market demand for this algal strain; these factors were also considered during the algal strain selection.

In accordance with the manufacturer’s guidelines, 1.87 g of a nutrient mixture was added to 1 L of the used algae suspension to achieve a dark green color within a period of 7–15 days. The composition of the applied nutrient solution (pH = 7.0 ± 0.2 at 25 °C) is summarized in Table 2. The cultivation of *C. vulgaris* was performed in Erlenmeyer flasks at a determined temperature (25 ± 1 °C) and light conditions (continuous illumination by 4 Osram Biolux [T8 L30W/965 Daylight 6500K 26 × 895 mm] full-spectrum fluorescent tubes emitting daylight).

Based on the methodology described by Vince Ördög [126], the experimental flasks were positioned on a glass shelf. A set of fluorescent tubes was placed beneath the glass shelf to ensure uniform illumination of the flasks. The applied arrangement allows the maximum surface area of the flasks to be illuminated from below, optimizing light exposure conditions. Aeration of the flasks was achieved using a Hailea ACO-9610 air pump (Hailea Co., Chaozhou, China). The generated microbubbles were diffused through an airstone, ensuring effective oxygenation of the culture medium. Before entering the flasks, the air was filtered using a polyvinyl difluoride (PVDF) syringe filter with a pore size of 0.22 μm (d = 13 mm). This filtration step helped maintain a sterile and controlled experimental environment.

### 2.2. Determination of Algal Density

During the measurement of algal biomass using the selected methods, a dilution series was prepared from a stock culture of *C. vulgaris.* The cell concentration in the stock culture was 1.08 × 10^11^ cells/L, and the stock was diluted with increasing ratios of 2, 5, 10, 20, 50, 100, 200, 500, and 1000. To allow comparative assessment of the UV–Vis spectrophotometric method, algal biomass was determined by seven different methods, including filtration, evaporation, Chl a extraction, determination of Mg concentration, and cell count, in addition to fluorescence detection and UV–Vis spectroscopy. The UV–Vis spectra were measured only in two parallels due to the negligible variances (R^2^ > 0.999). Cell counting was performed in four replicates on each member of the dilution series to ensure adequate representativeness. Except for the Mg content, all parameters were measured within 24 h to avoid possible temporal changes due to the living nature of the sample.

#### 2.2.1. Determination of Algal Biomass by Filtration

The algae suspension was filtered through sterile membrane filters (Whatman 10401170, Maidstone, UK, cellulose nitrate membrane, pore size = 0.45 μm, d = 47 mm) that were previously washed, dried, and weighed. The filtered cells were dried at 105 °C for 24 h, and the weight of the filters with the dried cells was remeasured on an analytical balance. To ensure the accuracy of the measurement, the filter paper with the dried algae was returned to the drying cabinet for another 3 h, confirming that the measurement was indeed based on mass constant.

#### 2.2.2. Determination of Algal Biomass by Evaporation

The algae suspensions at different dilutions were homogenized by shaking in volumetric flasks before being aliquoted into crucibles in triplicates (3 × 40 mL). The aliquots were then evaporated at 90 °C to constant weight in an oven, and residual weights were measured.

In parallel experiments, the algal suspensions were centrifuged in triplicates at each dilution in an Ohaus Frontier 5706 centrifuge (Ohaus Corporation, Parsippany, NJ, USA) at 5000 rpm for 20 min. The supernatants were decanted from the pelleted algae biomass, filtered through fluted membrane filter disks (Whatman 10401170, pore size = 0.45 µm, d = 47 mm) to eliminate any residual particles, and then evaporated at 90 °C to constant weight in an oven. The average residual weights per volume of the algae-free supernatants were calculated and averaged in triplicates and subtracted from the average algae concentrations determined in triplicates by evaporation without filtration. Thus, dissolved salt content potentially originated from the applied nutrient solution was deducted from the overall residual weight in each case. Such correction for the residual weight of nonalgae origin was carried out separately at all dilution rates examined.

#### 2.2.3. Measurement of Chlorophyll a Concentration and Magnesium Content

Spectrometric determination of the Chl a concentration in the samples was performed with the use of the Felföldy method [127]. The applied method uses methanol to extract Chl a pigment from the algae cells [115,128]. After a sonication process, the extracted Chl a was separated from the sample matrix through centrifugation (at 4700 rpm for 20 min). The spectrophotometric analysis of the Chl a content of the samples was performed by Jenway 6405 UV/VIS Spectrophotometer (Cole-Parmer Instrument Co., Neots, UK) at different wavelengths (750, 666, and 653 nm). The Felföldy formula was used to determine the Chl a content [µg/L] in the samples [127].

During the determination of Mg concentrations, samples of the biomass residues after evaporation (see Section 2.2.2) were subjected to ignition. Subsequently, Mg content was determined from the ash after the acid hydrolysis method [129,130] using a microwave-induced plasma atomic emission spectrometer 4210 (MP-AES Agilent Technologies, Santa Clara, CA, USA) for concentration termination.

#### 2.2.4. Determination of Cell Count

**Figure 1:**
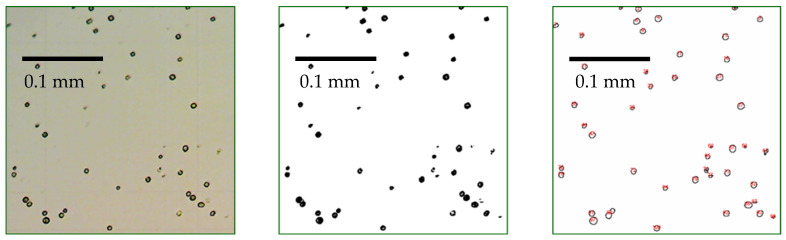
Cell counting from a microscopy image using imaging software: microscopy image (**left**), microscopy image after background subtraction process (**middle**), and after cell counting process by ImageJ (**right**).

Algae samples were added to test tubes in duplicates from each flask containing the various dilutions. Two pictures were taken of each sample with the use of a BTC BIM 105M microscope (Castell Nova Kft., Sopron, Hungary) coupled with a MicroQS 2-megapixel SPCMOS02000KPA microscope camera (Micro Q Inc., San Diego, CA, USA) at magnifications of 100× and 400×. The results are obtained from the average of 4 repetitions of image processing to ensure the accuracy and reliability of the method. The number of cells was determined by counting with the use of the Bürker counting chamber (Marienfeld-Superior, Lauda-Königshofen, Germany). From each microscope image, a specific area measuring 0.64 mm^2^ was selected for detailed image analysis and subsequent cell counting. ImageJ imaging software (software version number: 1.50i) was used to count the cells after performing image processing steps on the microscopic images. Firstly, a background subtraction process was applied with a light background. The images were then converted to 8-bit-type and underwent threshold adjustment to enhance contrast and convert them into black and white format, with a threshold range set from 0 to 200. These initial three steps were aimed at eliminating unwanted background noise and enhancing the visibility of the algae cells on the glass surface. Subsequently, the particles present in the images were analyzed using the “Analyze particles” command, considering a size range of 6150 pixels. This step ensured that only cells within the desired size range were counted while disregarding any potential smaller or larger contaminants. The resulting images, including the original microscopic images of the cells and their noise-filtered and edited versions, as well as the images after the counting process, can be observed in Figure 1. These processed images provide a clearer visualization of the algae cells and facilitate accurate cell counting and analysis.

#### 2.2.5. Estimation of Algal Biomass Based on the Detection of Induced Chlorophyll a Fluorescence

The algal biomass of the samples was determined by the prototype of a novel instrumentation, the Dichroic Fluorometer System (DFS), with a sample holder designed to fit 96-well ELISA microplates, developed within the framework of Project Aquafluosense (NVKP_16-1-2016-0049) [29]. The DFS was developed for algal density estimation and composition analysis based on the induced fluorescent excitation of Chl a. The measurements were performed according to the previously optimized and described method with the detection of fluorescence intensity expressed in relative fluorescence units (RFUs) [29]. The DFS was equipped with stepping motors to move both detector heads across the 96-well microplates, which ensured a fast, effective, and individual determination of RFUs in each well of the microplate [29]. During the measurements, RFUs were determined by Channel 1 (excitation wavelength: 630 nm, detection wavelength: 716 nm) of the developed DFS. The fluorescent excitation of Chl a was induced by LED as an actinic source [29]. During the measurements, algal cells were kept in the algal medium used to maintain the cultures and prepare the algal dilution series.

#### 2.2.6. Determination of Algal Optical Density by Spectrophotometry

The optical density was determined by spectrophotometric scanning at the wavelength range of 300–1100 nm using a Jenway 6405 UV/VIS Spectrophotometer (Cole-Parmer Instrument Co., Neots, UK). Due to the extremely low standard deviation of the measured spectra data lines (R^2^ > 0.999 based on linear regression analysis), only two repetitions were conducted for each dilution. To obtain corrected spectra, a blank spectrum of distilled water was obtained, and its absorbance values were subtracted from the average absorbance values of the samples.

### 2.3. Data Analysis

The results of the different analytical measurements were visualized and analyzed with the use of Microsoft Excel version 2018 (Microsoft Corporation, Redmond, WA, USA). Correlations between algae concentrations and UV–Vis absorbances at individual wavelengths between 190 and 1100 nm were determined, and R^2^ values of the correlation at each wavelength were determined by the use of an in-house software in Borterres Delphi^®^ 6.0 for Microsoft^®^ Windows 98^®^ and higher operating systems, developed by Imre Czinkota. The source code of the software can be found in the Supplementary Material. The essence of the calculation is that the program performs a stepwise sequence of linear regression analyses for the concentration–absorbance function at each wavelength individually and performs this analysis at each wavelength in 1 nm steps (910 correlation calculations per type of algae suspension). The resulting cross-section, slope, and correlation coefficient values are recorded in a table as a function of the wavelength. Due to absorbance saturation phenomena in the UV wavelength range below 300 nm, wavelength data were plotted and assessed at the 300–1100 nm range. The results stored in the obtained table can be further evaluated with the usual statistical methods.

The optical density values were used on a selected wavelength for further investigations. The selection of the wavelengths was based on the highest R^2^ values obtained from the linear regression scanning carried out by the program (see Section 3.2). Correlations among absorbance and values obtained in the parallel biomass determination methods were analyzed statistically using built-in functions in Microcal Origin 6.0 (MicroCal Software, Inc., Northampton, MA, USA), and the graphs presented were also created in this software.

## 3. Results

### 3.1. Results of Laboratory Measurements Used for the Determination of Algal Density

The average of the measured parameters obtained from the selected biomass determination methods performed on the same set of samples are summarized in Table 3. For the algal biomass measured by evaporation (BMe), the results below the 100× dilution were excluded from the correlation analyses, while for the biomass values obtained from filtration (BMf), only the results of the 1000× dilution were omitted, as they were under the limit of detection (LOD). In the case of the measurement of Mg content, it is likely that below the 20× dilution, after ignition, so little material was available that all of these values were under the LOD. However, in this case, we did not exclude these results because they do not significantly alter the observed trend.

Based on the summary table of correlations obtained from the results of parameters measured simultaneously on each member of the dilution series, the numbers represent the highest R^2^ values obtained by the best fitting trendlines. The remarkably high correlation values (R^2^ ≥ 0.990) are highlighted in bold letters. Table 4 does not contain the correlation analysis for spectroscopy, since these relationships are analyzed separately below.

### 3.2. Coefficient of Determination Correlation Scanning on the Whole Spectra

**Figure 2:**
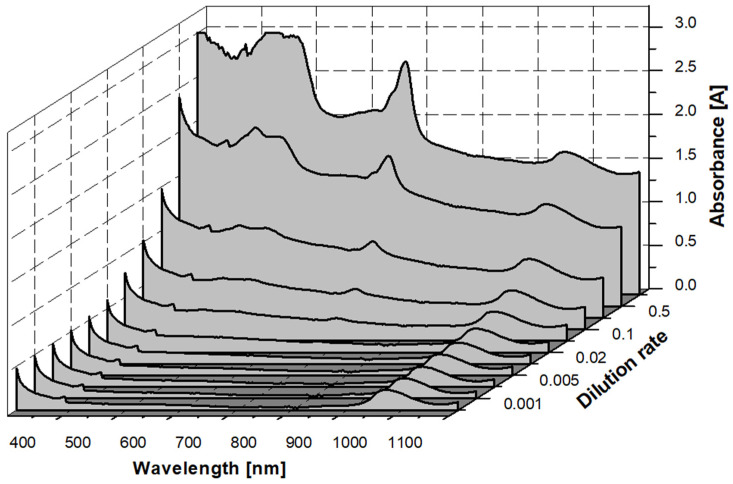
Measured spectra of *Chlorella vulgaris* at different dilution levels.

In addition to the fact that the correlations presented above may be important in the interoperability of individual parameters that can be measured in the laboratory, the main focus of our article is the correlations between these parameters and optical density. To present the measured differences in light intensity across the 300–1100 nm spectrum, the different dilutions were placed next to each other (Figure 2). The shape and peaks of the measured spectra are primarily influenced by the chlorophyll content of the algae [131], and the intensity of the signal deviations is presumably related to the algae concentration.

Due to the dilution series, the spectra shown in the graph above produce a series of 10 data points at each wavelength. These data sets can be individually compared with the results of the laboratory algae concentration measurements from the same 10 samples from the dilution series. To determine the best correlation of each of these parameters on the UV–Vis spectrum, we would have to fit a function with a trend line to the two data sets at each point. This would mean a total of 800 functions per parameter. The Delphi-based program was written to automate this task, which continuously scans the R^2^ values indicating the strength of the linear regression relationships.

**Figure 3:**
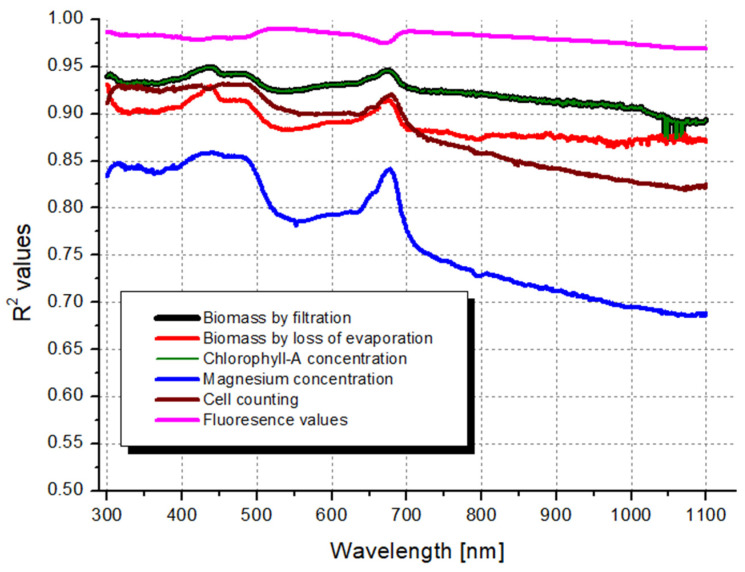
Strength of the R^2^ correlations between the optical density (absorbance) measured for the dilution series at each wavelength and the individual measured parameters on the entire spectrum.

Based on the results of correlation scanning implemented on the complete measured spectra, the higher values represent the higher correlation which showed fluctuations at the different wavelengths and local maximum values where the original spectra also had peak values (Figure 3). The only exception was observed in the case of fluorescence values, where roughly the opposite was observed. The lack of the curve characterizing BMf (colored in black) might be explained by the outstandingly strong correlation between BMf values and the Chl a concentration data shown in green color (see Section 4.2).

Since most of the parameters exhibited their maximum values around the 440 nm wavelength, and many spectrophotometers are not capable of handling increments smaller than 5 nm, we propose using the relationship between the biomass weight estimated by loss of evaporation and the values measured at 440 nm as a general formula (see Section 3.3).

The measured parameters exhibit maximum correlation values in the visible (400–800 nm) wavelength range (Table 5). As expected, it makes more sense to measure where the algae absorb light more effectively, as higher correlation coefficients are observed in these regions. However, it was unexpected that the peak around 680 nm gave lower coefficients of determination in all cases, since this is a clear, easily discernible local maximum of the curve. Thus, this peak is often used in the literature for biomass estimation (Table 1). By the methodology of software-based correlation scanning of the highest coefficient of determination, our result can help a long-standing professional dispute on the most proper wavelength for spectroscopic algal biomass estimations finally come to an end.

### 3.3. Recommended Formula for Algal Biomass Estimation

**Figure 4:**
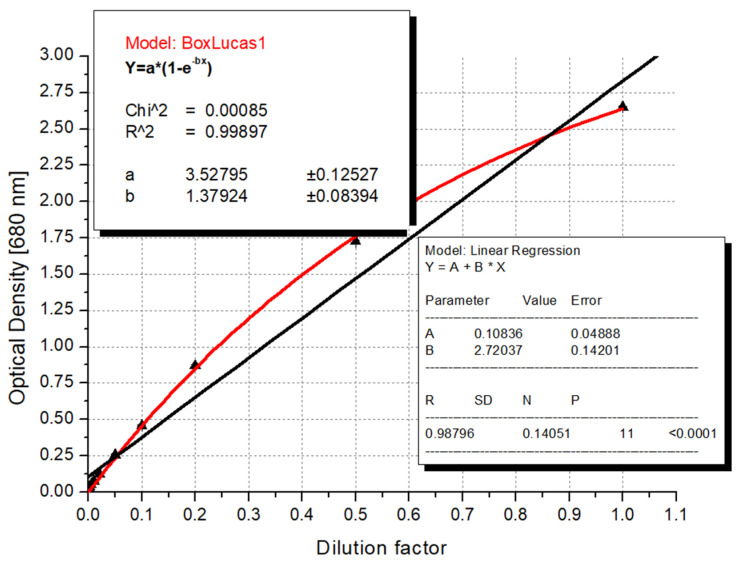
Comparison of accuracy of functions fitted with linear and Box–Lucas equations between the dilution factor and the optical density results measured at the 680 nm peak (red line: Box–Lucas fitting; black line: linear regression).

After the selection of the proper wavelengths, the correlation was sophisticated further by testing different built-in functions of the Origin software (version number 6.0). The parameters measured along the dilution series exhibited a sigmoid (logistic) curve when fitted to the values of optical density. This correlation can be explained by the phenomenon that, after a certain number of algae cells, there is a statistically high chance of covering each other, so the appearance of another cell will no longer cause as much of a drop in light intensity in a more concentrated suspension as it does in a dilute case. Therefore, for these parameters, we applied the Box–Lucas function (y = a·(1 − exp(−bx))) [132], which is a built-in factor of the Origin software and is equivalent to the well-known Bouguer–Lambert–Beer equation, which describes the light scattering effect during pigment determination [133]. As it is presented in Figure 4, the recommended logistic function resulted in a much higher coefficient of determination (R^2^ = 0.9989) than the linear model (R^2^ = 0.9879).

**Figure 5:**
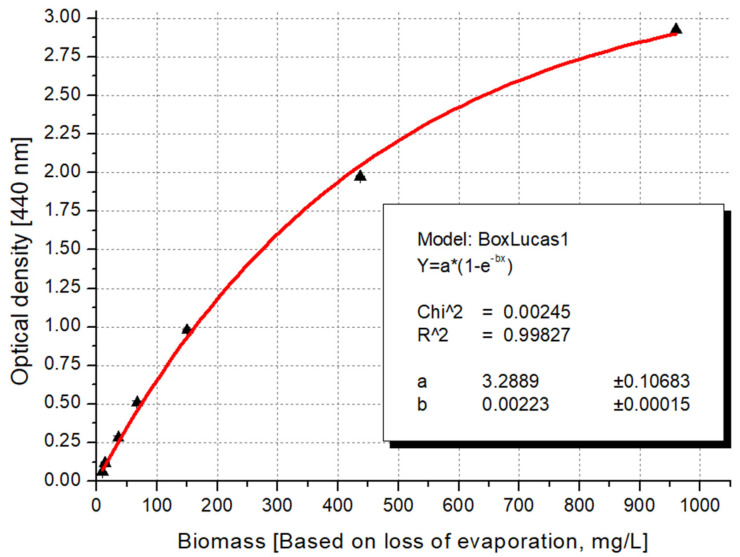
Optical density values correlation to dry algal biomass data determined by loss of evaporation method.

Since most of the parameters exhibited their maximum values around the 440 nm wavelength and many spectrophotometers are not capable of handling increments smaller than 5 nm, we propose using the relationship between the biomass weight estimated by loss of evaporation and the values measured at 440 nm as a general formula (Figure 5).

Algae biomass in mg (dry matter) = ln (1 − OD(440 nm)/3.29)/(−0.0022)(1)

### 3.4. The Mathematical Basis for Using the Logistic Curve

To characterize the parameters that exhibit a linear correlation, the equation from the Bouguer–Lambert–Beer law was applied in relation to optical density. This law states that if there is an obscuration phenomenon among particles, they proportionally reduce the magnitude of transmitted light. In this case, it is evident that if the algae absorb more light, it also obscures the light from the subsequent algae positioned behind it.

The theoretical background of this phenomenon can be explained as follows:

Let us assume that there is nothing else in the cuvette except algae cells neatly positioned behind and obscuring each other, meaning that the algae concentration is at its maximum, c_max_. In this case, let the measurable maximum absorbance be A_max_. This does not necessarily imply complete darkness (approximately 3–4 absorbance units, depending on the instrument), as algae cells do not absorb all light. However, we can be certain that the cells completely shade and obscure each other in this case. It is recognizable to see that the shading of algae cells is proportional to the concentration [134]. Therefore, the higher the concentration, the smaller the effect of newly added algae on absorbance. Approaching the maximum concentration, this effect becomes negligible, as the newly added algae are essentially shadowed by the others and do not contribute to further light absorption.

Expressed in the form of a differential equation, the decrease in light absorption as a function of concentration is proportional to the difference between the maximum measurable absorbance:

(2) $$\frac{dA}{dc}=-k\cdot (A_{max}-A)$$

where

*A*: Absorbance, [ ];
*c*: Concentration of algae, [mg/dm^3^, on dry base];
*k*: A specific light absorption coefficient characteristic of the given algae, [ ];
*A_max_*: The maximum absorbance characteristic of the given algae, [ ].

Let us introduce the following variable:

*B* = *A_max_* − *A* (3)

Since adding or subtracting a constant does not affect the change in a value, we can write: *dA* = *dB*. Substituting this into Equation (1):

(4) $$\frac{dB}{dc}=-k\cdot B$$

rearranging:

(5) $$\frac{dB}{B}=-k\cdot dc$$

This is a separable differential equation, with a known solution:

(6) ln(B)=−k·c+ln(K)

where *K* is the integration constant

rearranging:

(7) $$B=K\cdot e^{-k\cdot c}$$

Let us consider the integration constant for the following initial condition:

$$c=0,\;A=0\;\left(\mathrm{distilled}\;\mathrm{water}\right),\;\mathrm{thus}\;\mathrm{the}\;\mathrm{initial}\;B_{0}=A_{max}\;;\;\mathrm{therefore},\;K=A_{max},\;\left(\mathrm{as}\;\mathrm{the}\;\mathrm{exponent}\;\mathrm{of}\;\mathrm{zero}\;\mathrm{is}\;\mathrm{one}\right)$$

substituting into Equation (6):

(8) $$B=A_{max}\cdot e^{-k\cdot c}\;\mathrm{and}\;A=A_{max}-B$$

expressing *A* in terms of *A_max_*:

(9) $$A=A_{max}-A_{max}\cdot e^{-k\cdot c}$$

rearranging

(10) $$A=A_{max}\;\left(1-e^{-k\cdot c}\right)$$

The specific light absorption coefficient (or phytoplankton absorption coefficient) may be estimated from data regarding the in situ pigment distribution characteristics of the given algal species and their in vivo weight-specific absorption properties. The latter may be obtained through spectrophotometric measurement of the absorption characteristics of pure pigment standards, followed by adjusting their absorption maxima to correspond to in vivo conditions [135,136,137,138].

## 4. Discussion

### 4.1. Possible Areas of Application of the Results of Our Study

Due to the wavelengths determined by correlation scanning, and the further correlation analysis with the logistic curve, it is possible to carry out quick determination procedures in the algae production industry without damaging the sampled biomass. However, it still can be a question of which method is worth applying: fluorescence detection or optical density via UV–Vis spectrophotometry. The water testing standard ISO 7027-1:2016 recommends the use of two measuring instruments according to the number of polluting particles. For less contaminated samples (water purification), nephelometric measurement is used, while for highly contaminated, turbid samples (WWT), turbidimetric methods are applied [139,140].

**Figure 6:**
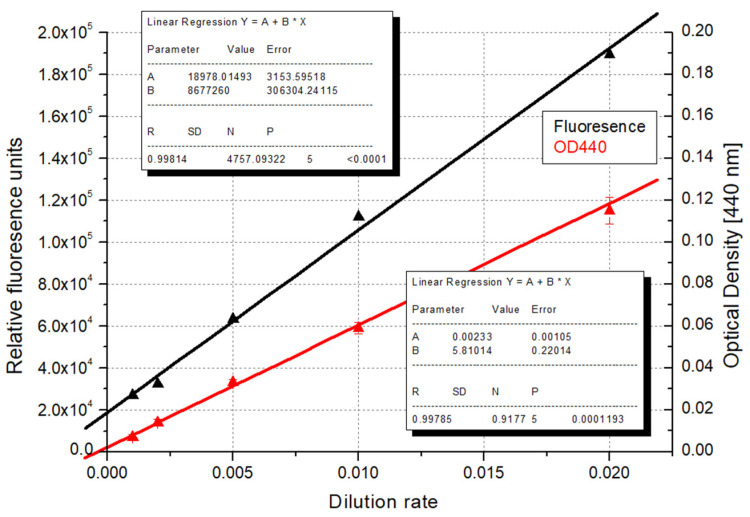
Correlation of dilution rates with optical density and fluorescence detection in the case of more diluted algae samples (OD—optical density; red line: linear regression based on the dilution rate and relative fluorescence values; black line: linear regression based on the dilution rate and optical density measured at 440 nm).

Using this assumption, we separated our 10 dilution samples into 5 more diluted and 5 denser sample groups and thus compared the strength of the correlations obtained for our data series. In the case of the more diluted samples, the data set measured by fluorescence detection resulted in a higher coefficient of determination (Figure 6). This might be explained by the operating principle of the measuring instrument. In this device, the light source–sample–detector form a 90-degree angle; thus, the scattered light at the specific angle is measured, which is highly effective when only a few algae particles are present in the sample.

**Figure 7:**
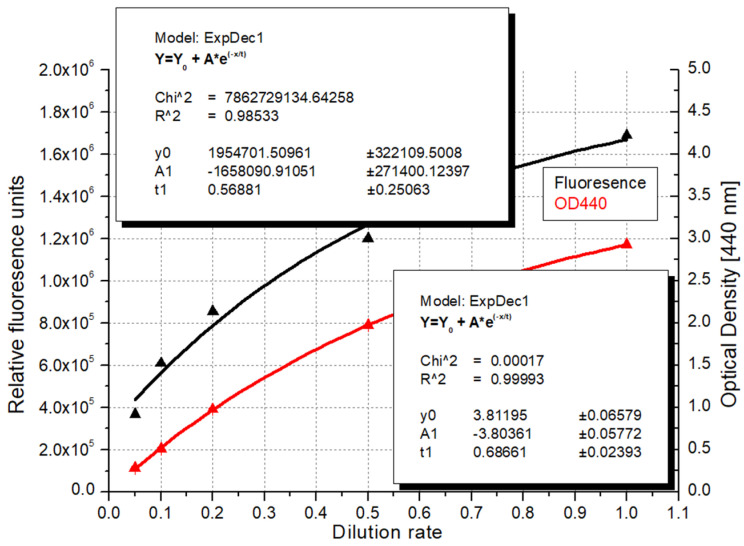
Correlation of dilution rates with optical density and fluorescence detection in the case of less diluted algae samples (OD—optical density; red line: exponential fitting based on the dilution rate and relative fluorescence values; black line: exponential fitting based on the dilution rate and optical density measured at 440 nm).

Not surprisingly in the case of the less diluted, more concentrated samples, the UV–Vis spectroscopy method generated more accurate results (R^2^ = 0.99993 compared with R^2^ = 0.98533 resulting from fluorescence (Figure 7). In this device, the light source–sample–detector form a 180-degree angle, with all three objects aligned, and thus, the absorbed light is measured. This arrangement makes it possible to achieve higher accuracy when the particles are more concentrated in the suspension.

### 4.2. Biomass Determination: Filtration or Loss of Evaporation

**Figure 8:**
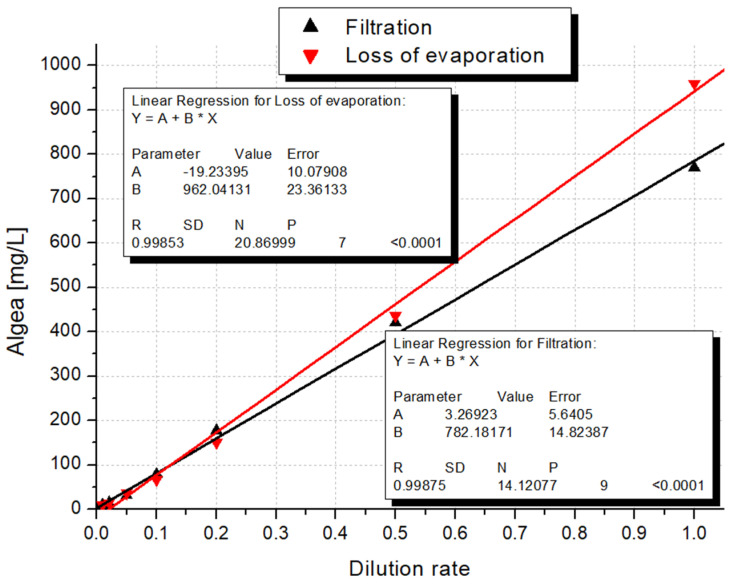
Correlation of dilution rate with dry algal biomass determined by loss of evaporation and filtration methods.

Despite the high difference observed in the case of maximum values (almost 20%), we can achieve very similar results regardless of which method we apply. The calculated R square value based on the linear correlation between biomass determined by filtration and the dilution rate values was 0.99875. Meanwhile, the coefficient of determination in terms of data loss of the evaporation method resulted in 0.99853 (Figure 8).

**Figure 9:**
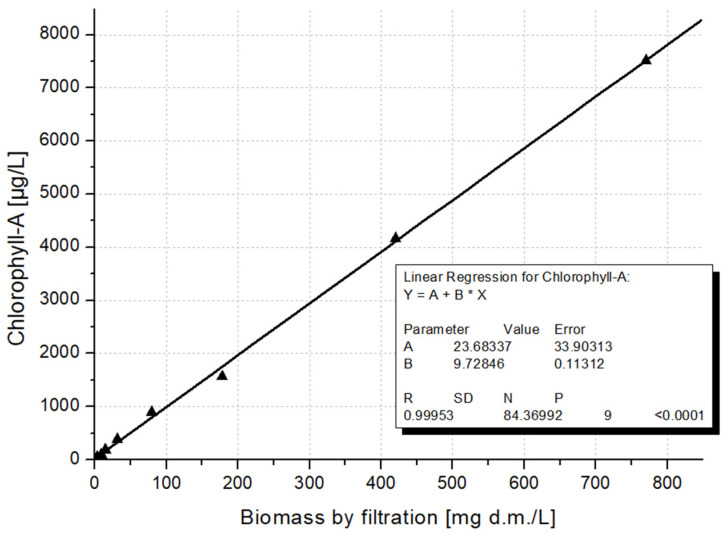
The strong correlation found between biomass values by filtration with the chlorophyll a values.

The correlation between the two result sets of biomass values by filtration to the Chl a values was outstandingly high: R^2^ = 0.99953. As a result, we obtained almost identical curves in the correlation scanning as well. This could be due to the filtering operation applied in the Felföldy method, which resulted in similar errors in both measurements (Figure 9).

### 4.3. Novel Automatized Method for Cell Counting Procedure and New Values for Cell Weight and Magnesium Content of Chlorella vulgaris

During the measurements, a rapid and partially automatized method was developed for the algae cell counting procedure based on the Image J software pixel counting feature. The detailed steps of the procedure are described in a previous section (see Section 2.2.5).

The average mineral Mg content of *C. vulgaris* algae can be derived from the measured biomass weight and Mg concentration data; meanwhile, the value of the average single-cell weight of this strain can be calculated from the biomass weight and cell number values. For these calculations, we generated an average value from the different dilutions. Due to the assumed inaccuracies of laboratory measurements at higher dilution levels, samples above 20-fold dilution did not form the basis of the calculation (Table 6).

Based on this calculation method, our value for the weight of 1 million *Chlorella* cells is 6358 μg ± 1542 μg or (6.358 ± 1.542) × 10^−10^ g/cell. In the scientific literature, it is surprisingly difficult to find data to compare our results. Based on the only available data on the topic, the recommended weight of a million cells was 22.4 μg ((2.24 ± 0.16) × 10^−11^ g/cell) [141], which approximates our measured value well in terms of magnitude.

As for the Mg content of *C. vulgaris*, our measurement resulted in a value of 20.156 ± 3.98 mg/100 g of dried algal biomass. Comparing this with the literature, an interesting scientific situation arose: the various authors can be categorized into two groups that differ in magnitude from each other (Table 7).

Since our determined value was found to be closer to only one published value [142], the only author who suggested a value of less than 100 mg/100 g dry matter, it was necessary to validate our value. For this reason, the Chl a content measured by the Felföldy method was compared with the theoretical, calculated values of Chl a content. In this calculation, the measured Mg values were multiplied by the ratio of 36.77, which is derived from the molar atomic mass of Mg (24.3 g/mol) and the molar molecular mass of Chl a (893.5 g/mol). As shown in Table 6, in the case of the undiluted samples, the calculated value (7521 μg/L) was almost equivalent (with a difference of 0.066%) to the measured value (7516 μg/L). Since our Mg measurement was validated by the previous deduction, and our biomass measurement was also validated by the fact that it matches the biomass data from the filtration method, we can conclude that our Mg content value from these two data sets must also be correct.

## 5. Conclusions

In our study, a novel linear regression scanning program was implemented to identify the optimal wavelengths for quick spectroscopic estimation of different parameters used to determine algal cell concentration. Through this innovative approach, the optimal wavelengths at which each parameter could be accurately estimated were successfully determined by the novel method of correlation scanning. Subsequently, we derived formulas for spectrometry-based estimation processes for each of these parameters. These formulas, coupled with the recommended optimal wavelengths, enable rapid determination procedures within the algae production industry without causing damage to the sampled biomass. Moreover, a general, nonlinear formula was also developed primarily for the quantitative estimation of algal biomass concentration, which serves as a foundational tool in algae production processes. Additionally, we provided recommendations for selecting measuring devices suitable for different algae concentration levels, considering the variations encountered during various stages of large-scale cultivation in high-rate algae ponds (HRAP) and photobioreactors. Our investigation also led to the establishment of new values for the Mg content and the average single-cell weight of the scientifically and economically preferred microalgae *C. vulgaris*. Furthermore, a rapid and partially automated method was developed for algae cell counting during our measurements based on the pixel counting feature of Image J software. This approach enhances efficiency and accuracy in quantifying algae cell populations, thereby facilitating comprehensive analysis in algae cultivation and biotechnology applications.

## Figures and Tables

**Table 1:** Summary of the spectroscopic methods for the estimation of the alga biomass of *Chlorella vulgaris* and the observed linear correlations between optical density and algal density.

Recommended Wavelength	R^2^	References
435 nm	0.980	[107]
440 nm	0.998	[108]
450 nm	n.a. ^a^	[109]
600 nm	n.a.	[110]
670 nm	n.a.	[111]
680 nm	n.a.	[112]
680 and 750 nm	n.a.	[101]
695 nm	0.970	[113]

^a^ not available.

**Table 2:** Composition of the culture medium.

Nutrient Solution Component	Concentration (g/L)
sodium nitrate	1.000
dipotassium phosphate	0.250
magnesium sulfate	0.513
ammonium chloride	0.050
calcium chloride	0.058
ferrous chloride	0.003

**Table 3:** The average of the measured parameters characterizing the algal density based on the selected biomass estimation methods.

Dilution	Ratio ^a^	BMe ^b^ (mg d.m. ^c^/L)	BMf ^d^ (mg d.m./L)	Chl-a ^e^ (μg/L)	Mg ^f^ (μg/L)	CN ^g^ (10^6^ cells/L)	RFU ^h^
1	1	960.0	770.0	7515.6	204.5	108,222.7	1,689,562
2	0.5	436.7	420.0	4163.1	109.6	61,578.1	1,200,521
5	0.2	150.0	177.5	1567.0	27.6	34,113.3	853,994
10	0.1	66.7	79.3	892.4	10.6	13,136.7	610,195
20	0.05	35.8	31.4	382.1	28.4	10,664.1	369,087
50	0.02	14.2	15.0	182.3	13.8	3757.8	189,411
100	0.01	10.0	9.3	90.9	15.4	1699.2	112,247
200	0.005	<LOD ^i^	2.5	54.6	17.5	472.7	63,474
500	0.002	<LOD	1.4	20.1	11.0	371.1	32,556
1000	0.001	<LOD	<LOD	12.3	6.9	105.5	26,938

^a^ ratio of dilution; ^b^ biomass based on evaporation; ^c^ dry matter; ^d^ biomass based on filtration; ^e^ chlorophyll a concentration; ^f^ magnesium concentration; ^g^ cell number; ^h^ relative fluorescence units; ^i^ under the limit of detection.

**Table 4:** Established correlations between the measured parameters.

	Dilution	BMf ^a^	BMe ^b^	Mg ^c^	CN ^d^	Chl-a ^e^	RFU ^f^
Dilution	-	**0.998** (L)	**0.997** (L)	0.975 (L)	0.986 (L)	**0.998** (L)	0.989 (Lg)
BMf	-	-	0.989 (L)	0.966 (L)	**0.993** (L)	**0.999** (L)	0.987 (Lg)
BMe	-	-	-	0.982 (L)	0.974 (L)	**0.990** (L)	0.974 (Ep)
Mg	-	-	-	-	0.945 (L)	0.971 (L)	0.816 (Ep)
CN	-	-	-	-	-	0.989 (L)	0.984 (Lg)
Chl-a	-	-	-	-	-	-	0.986 (Lg)
RFU	-	-	-	-	-	-	-

^a^ biomass based on filtration; ^b^ biomass based on evaporation; ^c^ magnesium content; ^d^ cell number; ^e^ chlorophyll a concentration; ^f^ relative fluorescence units; linear (L), logarithmic (Lg), or exponential (Ep) functions. Bold numbers indicate a high correlation between the measured parameters (R^2^ ≥ 0.990).

**Table 5:** Wavelengths at which optimal correlations (highest R^2^ value) occurred on the spectrum.

	BMf ^a^	BMe ^b^	Chl-a ^c^	Mg ^d^	CN ^e^	RFU ^f^
max R^2^	0.950	0.929	0.950	0.859	0.933	0.990
R^2^ max wavelength	439	437	439	442	461	529

^a^ biomass based on filtration; ^b^ biomass based on evaporation; ^c^ chlorophyll a concentration; ^d^ magnesium content; ^e^ cell number; ^f^ relative fluorescence units.

**Table 6:** Specific values derived from measured algae concentration data.

Dilution	BMe ^a^ (mg d.m. ^b^/L)	Mg (μg/L)	Mg ^c^ (mg/100 g d.m.)	Chl-a ^d^ from Mg (μg/L)	Chl-a ^e^ (μg/L)	CN ^f^ (10^6^ cells/L)	AW ^g^ (μg/10^6^ cells)
1×	960	205	21.31	7521	7516	108,223	8.871
2×	437	110	25.09	4029	4163	61,578	7.091
5×	150	28	18.40	1015	1567	34,113	4.397
10×	67	11	15.83	388	892	13,137	5.075
Average			20.16 ± 3.98				6.36 ± 2.03

^a^ biomass based on evaporation; ^b^ dry matter; ^c^ magnesium content; ^d^ chlorophyll a concentration based on calculation from magnesium content; ^e^ chlorophyll a concentration; ^f^ cell number; ^g^ average weight of 1 million algal cells.

**Table 7:** Magnesium content of *Chlorella vulgaris* according to the results of other studies.

Mg ^a^ Content (mg/100 g d.m. ^b^)	Reference
37.3	[142]
344	[143]
360	[144]
360–800	[145]
440	[146]

^a^ magnesium content; ^b^ dry matter.

## Data Availability

The original contributions presented in the study are included in the article/Supplementary Material, further inquiries can be directed to the corresponding author.

## Supplementary Materials

The following supporting information can be downloaded at https://www.mdpi.com/article/10.3390/microorganisms12061050/s1, Supplementary Material S1: Delphi source code for correlation scanning.txt.
